# Large Sporadic Vestibular Schwannoma Causing Brainstem Compression and Obstructive Hydrocephalus in a Young Adult

**DOI:** 10.7759/cureus.104905

**Published:** 2026-03-09

**Authors:** Adebola O Adetiloye, Anim Asif, Olurotimi J Badero

**Affiliations:** 1 Internal Medicine, Harlem Hospital Center, New York, USA; 2 Interventional Cardiology, Iwosan Lagoon Hospital, Lagos, NGA; 3 Interventional Cardiology, Division of Cardio-Nephrology, Cardiac Renal and Vascular Associates, Jackson, USA

**Keywords:** brainstem compression, obstructive hydrocephalus, rare case report, sporadic mutation, vestibular schwannoma

## Abstract

Sporadic vestibular schwannomas are typically slow-growing, unilateral cerebellopontine angle benign tumors that occur in middle-aged and older adults and rarely present in individuals younger than 30 years. We describe the case of a 21-year-old man who presented with progressive unilateral hearing loss and tinnitus and was found to have a large vestibular schwannoma causing brainstem compression and obstructive hydrocephalus. Tumor analysis demonstrated a somatic mutation in neurofibromatosis type 2 (NF2), confined to the tumor, supporting a diagnosis of sporadic vestibular schwannoma in the absence of NF2 features. This case highlights the importance of early neuroimaging in patients with persistent or progressive unilateral audiovestibular symptoms.

## Introduction

Vestibular schwannomas, or acoustic neuromas, typically account for 6-8% of all intracranial tumors and the vast majority, approximately 80-90%, of cerebellopontine angle (CPA) tumors [1]. These tumors most commonly occur sporadically in middle-aged adults, presenting insidiously with hearing loss, tinnitus, and imbalance [2]. Their occurrence in individuals under 30 years of age is distinctly uncommon and often raises suspicion for underlying neurofibromatosis type 2 (NF2), particularly when associated with spinal neurofibromas [3]. What makes this case unique is that the patient, a previously healthy 21-year-old male, developed a large vestibular schwannoma in the absence of syndromic features, presenting with unilateral hearing loss and tinnitus, and progressing to brainstem compression with obstructive hydrocephalus. Such a presentation at this age, in a non-NF2 setting, is exceptionally rare and highlights an unusual and clinically significant manifestation of an otherwise indolent tumor. Moreover, clearly documented single-case reports with confirmed somatic NF2 mutations in this age group are scarce, highlighting the rarity of this presentation.

## Case presentation

**Figure 1:**
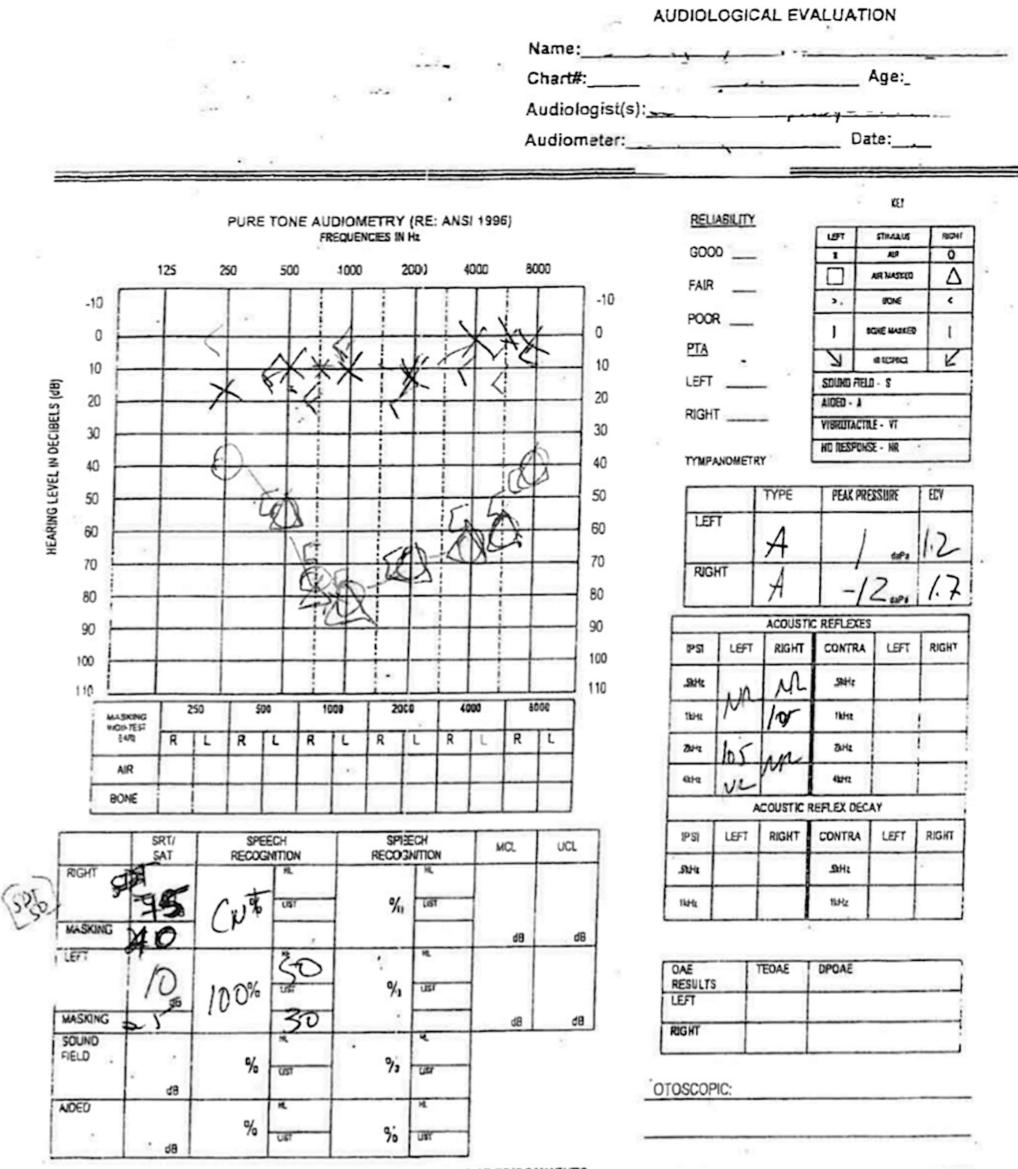
Pure-Tone Audiometry Pure-tone audiometry demonstrated mild-to-severe sensorineural hearing loss in the right ear across the 250-1000 Hz range. Hearing in the left ear was within normal limits.

A 21-year-old previously healthy Hispanic male presented with a two-year history of progressive right-sided hearing loss and intermittent tinnitus, which became prominent six months prior to presentation. He denied vertigo, facial numbness, or gait disturbance. Over the preceding two weeks, he reported worsening headaches, blurred vision, and nausea. Neurological examination revealed diminished right-sided hearing and a positive Rinne and Weber test lateralizing to the left. An ear examination showed a normal tympanic membrane. Audiometry confirmed right sensorineural hearing loss (Figure 1).

Fundoscopy showed papilledema, raising concern for increased intracranial pressure (Table 1).

**Table 1:** Fundoscopy Findings Funduscopic examination demonstrating bilateral grade 4 optic disc edema characterized by circumferential elevation of the disc margins and obscuration of major vessels in both eyes. The macula, retinal vessels, and peripheral retina are otherwise normal bilaterally.

Part	Right Eye	Left Eye
Disc	Grade 4 Edema	Grade 4 Edema
Macular	Normal	Normal
Vessels	Normal	Normal
Periphery	Normal	Normal

**Figure 2:**
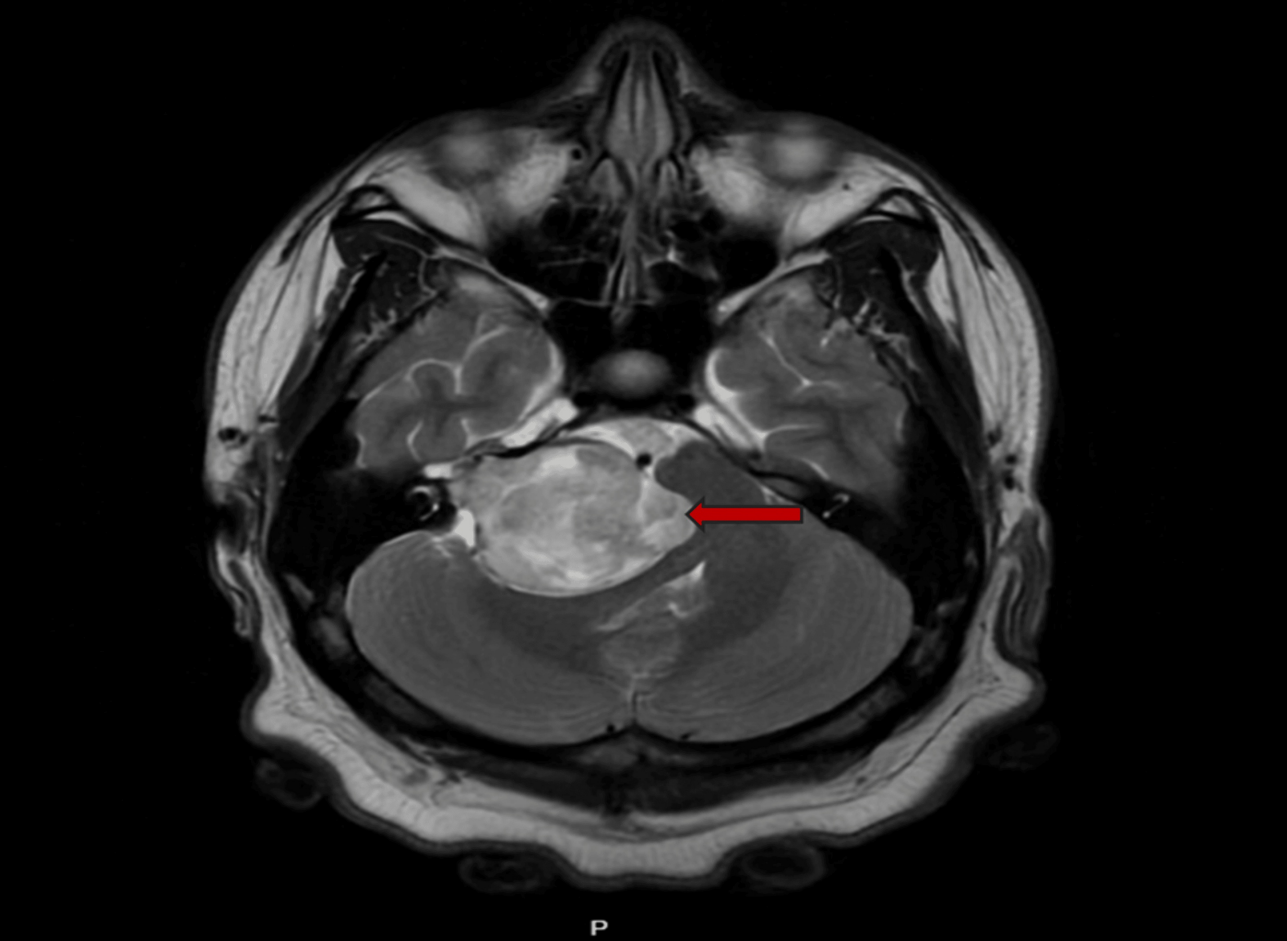
Magnetic Resonance Imaging (MRI) of the Internal Auditory Canal MRI of the internal auditory canal shows a homogenously high T2/FLAIR signal intensity mass centered within the right lateral cerebellopontine angle with direct extension into the right internal auditory canal. The mass measures approximately 4.9 x 4.2 x 4.2 cm (red arrow). There are mass effects and effacement of the lateral aspect of the pons and midbrain, along with near-complete effacement of the 4th ventricle with hydrocephalus. FLAIR: Fluid-Attenuated Inversion Recovery

**Figure 3:**
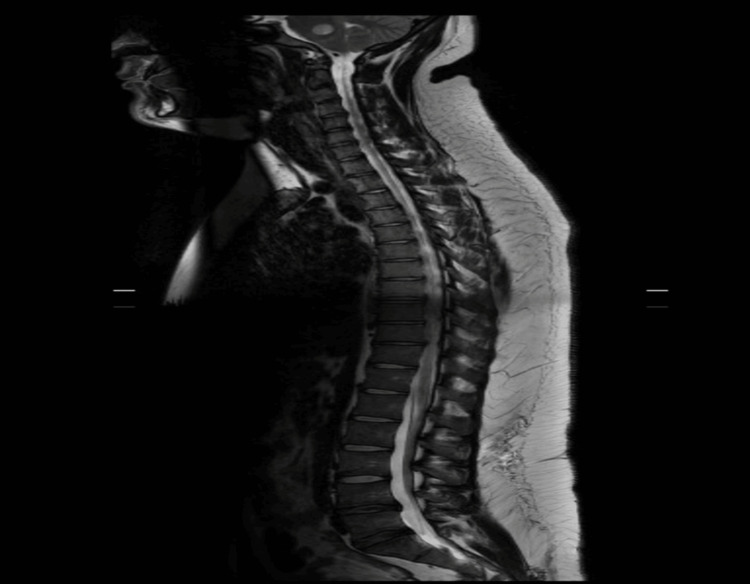
Sagittal Magnetic Resonance Imaging (MRI) of the Spine Sagittal MRI of the spine did not show evidence of leptomeningeal disease or drop metastasis.

Magnetic resonance imaging (MRI) of the brain revealed a 4.9 x 4.2 x 4.2 cm heterogeneously enhancing mass in the right cerebellopontine angle, extending into the internal auditory canal, consistent with a vestibular schwannoma. The mass compressed the adjacent pons and cerebellum, resulting in effacement of the fourth ventricle and dilatation of the lateral and third ventricles, consistent with obstructive hydrocephalus (Figure 2). MRI of the spine did not show spinal neurofibromas (Figure 3).

**Figure 4:**
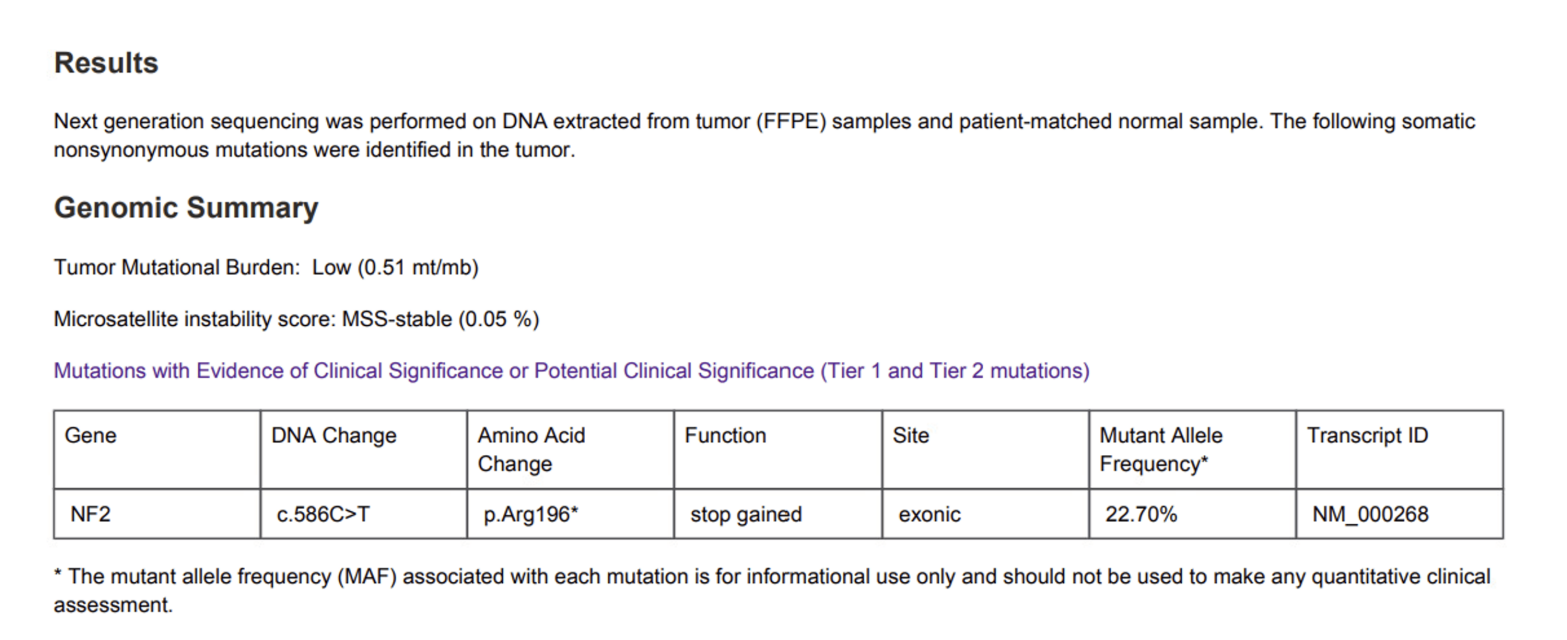
Tumor genomic profiling results Next-generation sequencing of tumor tissue with matched normal DNA identified a somatic NF2 c.586C>T (p.Arg196*) nonsense mutation, resulting in a premature stop codon and predicted truncation of the Merlin tumor suppressor protein. The mutation was present in tumor tissue but absent in the matched normal sample, consistent with a somatic (tumor-only) alteration.

The patient denies a family history of brain or spinal tumors. The patient underwent placement of an occipital ventriculoperitoneal (VP) shunt for cerebrospinal fluid diversion to alleviate hydrocephalus and a right transpetrosal approach for tumor resection three weeks later. Postoperatively, the patient recovered well with excellent gait and balance. The surgical specimen was confirmed as a DNA methylation-profiled schwannoma, classified as CNS WHO Grade 1, consistent with a benign, slow-growing neoplasm with a favorable prognosis. Genetic analysis of the tumor revealed a somatic non-synonymous NF2 mutation, supporting the loss of merlin tumor suppressor function. Importantly, this mutation was restricted to the tumor tissue and was not detected in the matched normal sample or peripheral blood, confirming its somatic origin (Figure 4). The patient was scheduled for follow-up with the neurosurgery and otolaryngology teams but missed the appointment.

## Discussion

Vestibular schwannomas are typically slow-growing and benign but may present with mass effect if left undiagnosed [3,4]. Most commonly, patients report unilateral sensorineural hearing loss and tinnitus, as seen in our case [5]. The median age at diagnosis is 55 years, with similar rates among males and females but higher in the Caucasian population [6]. A three-year retrospective epidemiological analysis of a national vestibular schwannoma registry linked to electronic patient records demonstrated that vestibular schwannomas are predominantly diagnosed in middle-aged and older adults [7]. The incidence among individuals younger than 40 years was low, ranging from 0.3 to 0.7 per 100,000 person-years, but increased substantially with age, reaching 5.7 to 6.1 per 100,000 person-years in those aged 60 to 69 years [7]. In another study, incidence ranged from 0.4 to 2.0 per 100,000 among patients aged 20-39 years to 9.9 to 11.1 per 100,000 among patients aged 50-69 years in the United States [8].

Sporadic vestibular schwannomas usually arise from somatic NF2 mutations confined to the tumor, while NF2-related schwannomatosis is characterized by a germline NF2 mutation present throughout all body cells [9]. This case is considered sporadic rather than associated with NF2 because the patient had no family history of NF2, no cutaneous stigmata, and no evidence of bilateral vestibular schwannomas or other intracranial or spinal tumors on imaging [9]. In our patient, the NF2 mutation was confined to the tumor (somatic) with no germline alteration, supporting a diagnosis of sporadic schwannoma. A study of 51 patients who underwent surgery for the removal of vestibular schwannomas reports NF2 mutations in approximately 49% of sporadic vestibular schwannomas [10].

Young adults presenting with isolated audiovestibular symptoms may not be immediately evaluated with imaging, leading to delays in diagnosis. Hydrocephalus occurs in approximately 3.7-42% of cases and is more common in tumors larger than 3 cm, due to compression of the fourth ventricle or cerebral aqueduct [11,12]. In our patient, the delayed diagnosis likely contributed to the tumor reaching a size sufficient to obstruct cerebrospinal fluid flow and cause significant brainstem compression. Early MRI is crucial in patients with unilateral sensorineural hearing loss or unexplained tinnitus, particularly if symptoms progress. The mainstay of treatment for large symptomatic tumors remains surgical resection, although radiosurgery may be considered in smaller lesions or poor surgical candidates [13].

## Conclusions

This case underscores the importance of early neuroimaging in young adults presenting with persistent or progressive audiovestibular symptoms. Vestibular schwannomas may occur sporadically in younger patients without features of NF2, and delayed recognition can result in significant morbidity and potentially life-threatening complications such as progressive brainstem compression and obstructive hydrocephalus.
